# Extracorporeal cardiopulmonary resuscitation with temperature management could improve the neurological outcomes of out-of-hospital cardiac arrest: a retrospective analysis of a nationwide multicenter observational study in Japan

**DOI:** 10.1186/s40560-022-00622-7

**Published:** 2022-06-17

**Authors:** Toshihiro Sakurai, Tadashi Kaneko, Shu Yamada, Takeshi Takahashi

**Affiliations:** 1 Emergency and Critical Care Center, Kumamoto Medical Center, Kumamoto, Japan; 2 Emergency and Critical Care Center, Mie University Hospital, Tsu, Japan

**Keywords:** Mild therapeutic hypothermia, Extracorporeal membrane oxygenation, Cerebral performance category

## Abstract

**Background:**

Target temperature management (TTM) is an effective component of treating out-of-hospital cardiac arrest (OHCA) after return of spontaneous circulation in conventional cardiopulmonary resuscitation. However, therapeutic hypothermia (32–34 °C TTM) is not recommended based on the results of recent studies. Extracorporeal cardiopulmonary resuscitation (ECPR) with veno-arterial extracorporeal membrane oxygenation is another promising therapy for OHCA, but few studies have examined the effectiveness of ECPR with TTM. Therefore, we hypothesized that ECPR with TTM could have the effectiveness to improve the neurological outcomes for adults following witnessed OHCA, in comparison to ECPR without TTM.

**Methods:**

We performed retrospective subanalyses of the Japanese Association for Acute Medicine OHCA registry. We focused on adults who underwent ECPR for witnessed OHCA. We performed univariate (the Mann–Whitney *U* test and Fisher’s exact test), multivariable (logistic regression analyses), and propensity score analyses (the inverse probability of the treatment-weighting method) with to compare the neurological outcomes between patients with or without TTM, among all eligible patients, patients with a cardiogenic cause, and patients divided into subgroups according to the interval from collapse to pump start (ICPS) (> 30, > 45, or > 60 min).

**Results:**

We analyzed data for 977 patients. Among 471 patients treated with TTM, the target temperature was therapeutic hypothermia in 70%, and the median interval from collapse to target temperature was 249 min. Propensity score analysis showed a positive association between TTM and favorable neurological outcomes in all patients (odds ratio 1.546 [95% confidence interval 1.046–2.286], *P* = 0.029), and in patients with ICPS of > 30 or > 45 min, but not in those with ICPS of > 60 min. The propensity score analysis also showed a positive association between TTM and favorable neurological outcomes in patients with a cardiogenic cause (odds ratio 1.655 [95% confidence interval 1.096–2.500], *P* = 0.017), including in all ICPS subgroups (> 30, > 45, and > 60 min).

**Conclusion:**

Within patients who underwent ECPR following OHCA, ECPR with TTM could show the potential of improvement in the neurological outcomes, compared to ECPR without TTM.

## Background

Target temperature management (TTM) after return of spontaneous circulation (ROSC) is an important topic in the treatment of patients with out-of-hospital cardiac arrest (OHCA). Therapeutic hypothermia (TH: 32–34 °C TTM) after ROSC with conventional cardiopulmonary resuscitation (CCPR) is not currently recommended in recent reviews [1, 2]. Although TH is now being considered, the ideal TTM has not been established yet and more research is needed [3]. It has also been suggested that, when using TH, faster cooling is important to improve the neurological outcome of OHCA [4, 5].

Extracorporeal cardiopulmonary resuscitation (ECPR) is a promising strategy for OHCA patients, especially when performed using veno-arterial (VA)-extracorporeal membrane oxygenation (ECMO). We previously reported that ECPR without TTM could prolong the therapeutic time window of OHCA compared with CCPR without TTM [6], and a shorter interval from collapse to target temperature (ICTT) in ECPR with TTM could improve the neurological outcomes of patients compared with CCPR with TTM [7].

During ECPR, the use of VA-ECMO could help to cool the patient more quickly than using conventional cooling methods, which rely on the spontaneous circulation. Therefore, it is possible that ECPR with TTM could provide additional effects in terms of improving the neurological outcome of OHCA, even with TH. However, there are no clear recommendations to suggest that TTM could provide an additional therapeutic effect to ECPR. (Our previous studies showed favorable neurological outcome, in ECPR with TTM: 17% [7], ECPR without TTM: 7% [6].)

Recent systematic reviews have shown a potential advantage of TTM following ECPR [8, 9], but the reviews had some limitations that affect the findings, such as the inclusion of in-hospital cardiac arrest cases, omission of the cooling rate, and absence of randomized controlled studies. Furthermore, the effect of TTM following ECPR for OHCA is unclear, especially when we consider the interval from collapse to pump start (ICPS), even though ECPR prolongs the therapeutic time window and improves the outcomes of TTM for OHCA, compare to CCPR from previous study results. Therefore, we hypothesized that ECPR with TTM could improve the neurological outcomes for adults following witnessed OHCA, compare to ECPR without TTM, with taking into consideration of ICPS.

In Japan, a nationwide observational registry of OHCA was established by the Japanese Association for Acute Medicine (JAAM), the JAAM-OHCA registry, in June 2014, that includes ECPR cases [10]. A retrospective analysis of OHCA cases registered between 2014 and 2019 was performed to investigate the effectiveness of ECPR with TTM. To assess the effectiveness of ECPR with TTM, the ICPS was considered to be a key factor to judge the eligible cases given favorable neurological outcome, although the study did not document the effectiveness of TTM among patients who underwent CCPR for OHCA. In the present study, we investigated whether ECPR combined with TTM, which prolongs the therapeutic window with faster cooling than CCPR, improves the neurological outcomes of patients with OHCA compared with ECPR without TTM.

## Methods

### Study design

In this study, we used data from the prospective JAAM-OHCA registry, which registered OHCA patients at 140 Japanese hospitals. The registry was approved by the ethics committees at Kyoto University, the participating institutions, and each participating hospital. We retrieved data for patients registered between June 2014 and December 2019 for retrospective analyses.

### Patients

Between June 2014 and December 2019, a total of 57,754 patients with OHCA were registered in the JAAM-OHCA registry. We retrieved data for patients aged > 18 years with witnessed OHCA who underwent ECPR combined with ECMO. Patients who died in the emergency department were excluded from the study.

### Study outcomes and statistical analysis

In this study, primary outcome was a comparison of favorable neurological outcome between the groups of ECPR for OHCA cases with and without TTM by propensity score analysis, which was one of the useful statistical methods to reduce biases among the groups.

The neurological outcomes were assessed in all patients using the Glasgow–Pittsburgh Cerebral Performance Category (CPC), which includes five categories: CPC 1 (good recovery), CPC 2 (moderate disability), CPC 3 (severe disability), CPC 4 (vegetative state), and CPC 5 (death) [11]. We defined a favorable neurological outcome as a CPC of 1–2 at 1 month after collapse.

In the present study, ECPR with TTM was defined as using cooling devices (surface or ECPR device cooling) and having TT to maintain, therefore, ECPR without TTM did not use any cooling devices. Intervals in ECPR with/without TTM were defined as interval from collapse to pump start (ICPS: interval from collapse of witnessed OHCA to ECMO pump start), interval from collapse to reach target temperature (ICTT), and interval of temperature management (ITM).

The patients’ age, sex, bystander cardiopulmonary resuscitation (BCPR), presence of a shockable rhythm (SR; ventricular fibrillation or pulseless tachycardia), ICPS, cardiogenic cause, ICTT, target temperature, ITM, and rate of temperature management started before or same time of ECMO pump start (TMPS) were retrieved from the database as potential confounding factors for analyzing the outcome of ECPR with TTM.

The patients were divided according to whether or not they received TTM and their neurological outcomes (favorable: CPC 1–2; unfavorable: CPC 3–5). The groups were compared using univariate and multivariable analyses. Univariate analyses comprised the Mann–Whitney *U* test or Fisher’s exact test, as appropriate. Multivariable analyses comprised logistic regression analyses with two models. In the first model (comparison of with and without TTM groups), the dependent variable was TTM and the independent variables were age, sex, BCPR, SR as the initial rhythm, ICPS, cardiogenic cause, and favorable outcome (CPC 1–2). In the second model (comparison of favorable and unfavorable outcome groups), the dependent variable was a favorable neurological outcome (CPC 1–2) and the independent variables were age, sex, BCPR, SR as the initial rhythm, ICPS, cardiogenic cause, and TTM. These variables were analyzed in all eligible patients.

Propensity score analysis was performed considering age, sex, BCPR, SR as the initial rhythm, ICPS, and cardiogenic cause using the inverse probability of the treatment-weighting (IPTW) method [12] to compare favorable neurological outcomes (CPC 1–2) between patients who received ECPR with or without TTM for all patients combined and in subgroups of patients with ICPS > 30, > 45, and > 60 min, to investigate upper limit of reperfusion time showing TTM effectiveness. Propensity score analysis with the IPTW method was also performed considering the age, sex, BCPR, SR as the initial rhythm, and ICPS among all eligible patients with a cardiogenic cause.

In all analyses, a *P* value of < 0.05 was considered statistically significant. All statistical analyses, except for the propensity score analysis, were performed with SPSS version 25.0 (IBM, Armonk, NY, USA). Propensity score analysis with the IPTW method was performed using R software version 4.0.1 (GNU general public license).

## Results

**Fig. 1:**
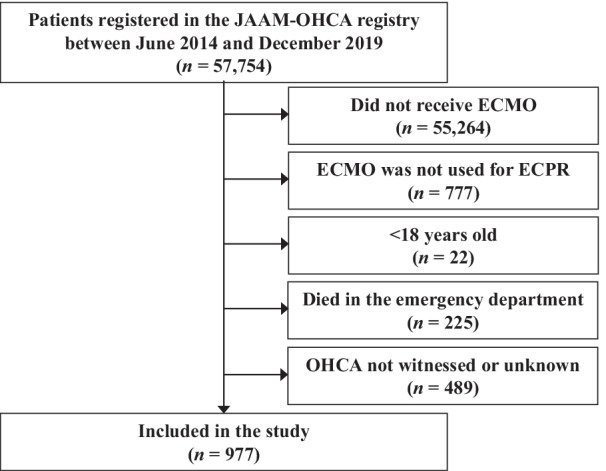
Patient disposition. A total of 977 OHCA patients were considered eligible after applying the inclusion criteria: ECMO, ECPR, age > 18 years, survival in the emergency department, and witnessed OHCA. *JAAM-OHCA* Japanese Association of Acute Medicine, Out-of-Hospital Cardiac Arrest, *ECMO* extracorporeal membrane oxygenation, *ECPR* extracorporeal cardiopulmonary resuscitation

The registry comprised 57,754 patients, of which 977 met the inclusion criteria (i.e., ECPR for witnessed OHCA, age > 18 years, and hospitalization; Fig. 1). Among 977 eligible patients, TTM was performed in 471; the other 506 did not receive TTM.

Table 1 shows the characteristics of patients who underwent ECPR with (*n* = 471) or without (*n* = 506) TTM. Multivariable analysis revealed significant differences between the two groups in terms of the age, cardiogenic causes, and favorable neurological outcomes. In the ECPR with TTM group, the median ICTT was 249 min and the TTs were 32 °C (1%), 33 °C (4%), 34 °C (65%), 35 °C (11%), and 36 °C (19%). Therefore, about 70% of patients were managed with therapeutic hypothermia, which was reached within 4 h. In ECPR with TTM patients, 100 cases (21%) were controlled by only surface cooling, and 189 cases (42%) were started temperature management before or at same time of ECMO pump start.

**Table 1:** Univariate and multivariable comparisons of patients who underwent ECPR with or without TTM groups

Variables	ECPR with TTM (*n* = 471)	ECPR without TTM (*n* = 506)	Univariate *P* value	Multivariable *P* value	OR (95% CI)
Age (years)	57 (47–67)	62 (49–71)	** < 0.001**	**0.001**	0.984 (0.975–0.993)
Male (%)	403 (86%)	425 (84%)	0.533	0.626	0.911 (0.626–1.325)
BCPR (%)	240 (51%)	234 (46%)	0.159	0.853	1.026 (0.786–1.338)
SR (%)	327 (69%)	319 (63%)	**0.036**	0.803	1.038 (0.777–1.386)
ICPS (min)	51 (42–62)	56 (46–69)	** < 0.001**	0.685	1.000 (0.999–1.001)
Cardiogenic cause	442 (94%)	422 (83%)	** < 0.001**	** < 0.001**	2.927 (1.818–4.710)
CPC 1–2 (%)	75 (16%)	50 (10%)	**0.005**	**0.025**	1.570 (1.058–2.330)
TMPS (%)	189 (42%)				
ICTT (min)	249 (105–400)				
TT (°C)					
32	6 (1%)				
33	18 (4%)				
34	302 (65%)				
35	51 (11%)				
36	86 (19%)				
ITM (h)	43 (26–50)				

*P* values < 0.05 were bolded

Values are median (interquartile range) or *n* (%) of cases

*TTM* target temperature management, *ECPR* extracorporeal cardiopulmonary resuscitation, *OR* odds ratio, *CI* confidence interval, *BCPR* bystander cardiopulmonary resuscitation, *SR* shockable rhythm, *ICPS* interval from collapse to pump start, *CPC* Glasgow–Pittsburgh Cerebral Performance Category, *ICTT* interval from collapse to reach target temperature, *TMPS* temperature management before or same time at ECMO pump start, *TT* target temperature, *ITM* interval of temperature management

Table 2 compares the patients with favorable (CPC 1–2) and unfavorable (CPC 3–5) neurological outcomes. The multivariable analysis revealed significant differences between the two groups in terms of their age, SR as initial rhythm, and TTM. The multivariable analysis showed that TTM (70% of patients with therapeutic hypothermia) during ECPR was positively associated with neurological outcomes (odds ratio [OR] 1.588 [95% confidence interval {CI} 1.072–2.354], *P* = 0.021).

**Table 2:** Univariate and multivariable comparisons of cases with favorable and unfavorable neurological outcomes

Variables	Favorable outcomes (CPC 1–2; *n* = 125)	Unfavorable outcomes (CPC 3–5; *n* = 852)	Univariate *P* value	Multivariable *P* value	OR (95% CI)
Age (years)	54 (45–65)	60 (49–70)	** < 0.001**	**0.001**	0.978 (0.965–0.991)
Male (%)	102 (82%)	726 (85%)	0.288	0.215	0.723 (0.432–1.208)
BCPR (%)	62 (50%)	412 (48%)	0.848	0.541	0.885 (0.597–1.311)
SR (%)	96 (77%)	550 (65%)	**0.006**	**0.013**	1.828 (1.138–2.937)
ICPS (min)	46 (36–56)	55 (45–66)	** < 0.001**	0.946	1.000 (0.998–1.002)
Cardiogenic cause (%)	113 (90%)	751 (88%)	0.550	0.836	0.928 (0.460–1.873)
TTM (%)	75 (60%)	396 (46%)	**0.005**	**0.021**	1.588 (1.072–2.354)

*P* values < 0.05 were bolded

Values are median (interquartile range) or *n* (%) of cases

*CPC* Glasgow–Pittsburgh Cerebral Performance Category, *OR* odds ratio, *CI* confidence interval, *BCPR* bystander cardiopulmonary resuscitation, *SR* shockable rhythm, *ICPS* interval from collapse to pump start, *TTM* target temperature management

Table 3 shows the results of the propensity score analysis using the IPTW method for comparing favorable neurological outcome (CPC 1–2) between patients who underwent ECPR with or without TTM in all patients and in subgroups of patients according to an ICPS of > 30, > 45, or > 60 min. TTM was positively associated with favorable neurological outcomes (CPC 1–2) in all patients (OR 1.546 [95% CI 1.046–2.286], *P* = 0.029), patients with an ICPS of > 30 min (OR 1.864 [95% CI 1.221–2.845], *P* = 0.004), and patients with an ICPS of > 45 min (OR 2.078 [95% CI 1.201–3.598], *P* = 0.009), but not in patients with an ICPS of > 60 min.

**Table 3:** Comparison of the proportions of patients with favorable neurological outcomes (CPC 1–2) between ECPR with or without TTM using propensity score analysis with the IPTW method

Variables	Treatment	*n*	CPC 1–2	OR (95% CI)	*P* value
All cases	ECPR with TTM ECPR without TTM	471 506	75 (16%) 50 (10%)	1.546 (1.046–2.286)	**0.029**
ICPS > 30 min	ECPR with TTM ECPR without TTM	441 475	70 (16%) 40 (8%)	1.864 (1.221–2.845)	**0.004**
ICPS > 45 min	ECPR with TTM ECPR without TTM	313 383	41 (13%) 24 (6%)	2.078 (1.201–3.598)	**0.009**
ICPS > 60 min	ECPR with TTM ECPR without TTM	123 199	13 (11%) 7 (4%)	2.686 (0.983–7.338)	0.055

*P* values < 0.05 were bolded

The propensity score analysis incorporated the following variables: age, sex, BCPR, SR, ICPS, and cardiogenic cause

*CPC* Glasgow–Pittsburgh Cerebral Performance Category, *OR* odds ratio, *CI* confidence interval, *ECPR* extracorporeal cardiopulmonary resuscitation, *BCPR* bystander cardiopulmonary resuscitation, *SR* shockable rhythm, *TTM* target temperature management, *ICPS* interval from collapse to pump start, *IPTW* inverse probability of the treatment-weighting

Table 4 shows the results of a similar propensity score analysis using the IPTW method limited to patients with a cardiogenic cause. In this analysis, TTM was positively associated with favorable neurological outcomes (CPC 1–2) in all patients (OR 1.655 [95% CI 1.096–2.500], *P* = 0.017), patients with an ICPS of > 30 min (OR 1.966 [95% CI 1.255–3.079], *P* = 0.003), patients with an ICPS of > 45 min (OR 2.356 [95% CI 1.312–4.233], *P* = 0.004), and patients with an ICPS of > 60 min (OR 4.544 [95% CI 1.233–16.741], *P* = 0.024, respectively).

**Table 4:** Comparison of the proportions of patients with favorable neurological outcome (CPC 1–2) between ECPR with or without TTM using propensity score analysis with the IPTW method among cases with a cardiogenic cause

Variables	Treatment	*n*	CPC 1–2	OR (95% CI)	*P* value
All cases	ECPR with TTM ECPR without TTM	442 422	72 (16%) 41 (10%)	1.655 (1.096–2.500)	**0.017**
ICPS > 30 min	ECPR with TTM ECPR without TTM	419 401	67 (16%) 32 (8%)	1.966 (1.255–3.079)	**0.003**
ICPS > 45 min	ECPR with TTM ECPR without TTM	302 327	40 (13%) 18 (6%)	2.356 (1.312–4.233)	**0.004**
ICPS > 60 min	ECPR with TTM ECPR without TTM	117 168	13 (11%) 3 (2%)	4.544 (1.233–16.741)	**0.024**

*P* values < 0.05 were bolded

The propensity score analysis incorporated the following variables: age, sex, BCPR, SR, and ICPS

*CPC* Glasgow–Pittsburgh Cerebral Performance Category, *OR* odds ratio, *CI* confidence interval, *ECPR* extracorporeal cardiopulmonary resuscitation, *BCPR* bystander cardiopulmonary resuscitation, *SR* shockable rhythm, *TTM* target temperature management, *ICPS* interval from collapse to pump start, *IPTW* inverse probability of the treatment-weighting

## Discussion

In the present study, the primary outcome of propensity score analysis of all eligible patients revealed that ECPR combined with TTM was associated with better neurological outcomes after OHCA compared with ECPR without TTM. In this study population, about 70% of cases underwent TTM shown as therapeutic hypothermia (32–34^0^C TTM). Although prior studies of CCPR cases have shown the effectiveness of therapeutic hypothermia [13, 14], these earlier studies used control groups without fever treatment, and controversially stated that the worse outcome was due to the onset of fever after ROSC, a topic that has been discussed recently [1, 2]. By comparison, ECMO during ECPR could prevent fever due to the heat dissipation effect of the extracorporeal circulation; thus, the control group of patients who did not receive TTM were unlikely to have developed high fever in this study, although the JAAM-OHCA registry did not record body temperatures during ECPR. Therefore, present study results might reveal the advantage of therapeutic hypothermia (70% of TTM group) compared to normothermia (fever treatment by heat dissipation).

We presume one of the advantages of ECPR with TTM is that it quickly lowers the body temperature constantly by directly lowering the blood temperature in the extracorporeal circulation instead of relying on spontaneous circulation. In this study, the median ICTT in patients who underwent TTM was 249 min. This rapid cooling may help improve the neurological outcome, as previously suggested (therapeutic hypothermia with within 3.5 h [4], around 200 min [5] of cooling period showed advantages). Furthermore, in subgroup analyses, patients with an ICPS of < 60 min, had better neurological outcomes than patients with a longer ICPS. In an analysis of cases with a cardiogenic cause, the effectiveness of TTM was also observed patients with an ICPS of > 60 min. Therefore, these results might suggest a benefit of TTM during ECPR in some ICPS conditions.

To confirm these possibilities of TTM benefits, and although ECPR with TTM still showed only 16% of favorable outcome, the indication for ECPR should be considered. Although one recent meta-analysis did not show positive outcomes of ECPR with TTM, this may be related to the heterogeneity of the study populations due to the broad use of ECPR [15]. To investigate the appropriate indications, a systematic review reported that the current standard indications of ECPR were age, witnessed OHCA, no flow (within 5–10 min), initial SR, and refractory cardiac arrest (10–30 min). In particular, patients with refractory cardiac arrest, defined as > 10 min, was associated with good neurological outcomes, which extended to those with refractory cardiac arrest of 15–30 min [16]. Most Japanese departments have similar protocols for starting ECPR, and our data showed positive neurological outcomes of ECPR with TTM. However, our data do not show the effectiveness of TTM in patients who undergo ECPR using other countries’ ECPR protocols, only showing outcome of ECPR in Japanese departments.

Overall, in this population of adult patients with witnessed OHCA, of whom about 90% had a cardiogenic cause, we have demonstrated the effectiveness of ECPR with TTM, which comprised therapeutic hypothermia in about 70% of patients, compared with ECPR in which heat was dissipated via extracorporeal circulation using VA-ECMO to prevent fever at least. In the majority of cases, the ICPS was less than 50 min and the median time to reach the target temperature was approximately 4 h. In previous studies of CCPR, there was no difference in positive outcomes between therapeutic hypothermia and normothermia [1, 2], despite positive effects of fever control versus uncontrolled fever [13, 14]. Our data suggest that therapeutic hypothermia with ECPR could improve the neurological outcomes of patients with OHCA. There are some possible reasons for our observations that should be discussed. First, the eligible population were generally not elderly, had an initial SR, and a cardiogenic cause, and early reperfusion was achieved by ECPR. Second, the time to reach the target temperature by ECPR with VA-ECMO was relatively short. Among cases of CCPR, rapid achievement of therapeutic hypothermia was a controversial finding [4, 5]. Third, the quick reperfusion and cooling of organs, not only the brain, might be beneficial. In CCPR, organ reperfusion started after ROSC, and relied on spontaneous circulation. By comparison, ECPR ensures early reperfusion and can help cool the body more quickly than CCPR can. This theory is supported by the results of animal studies in which quick cooling improved organ function after CCPR [17, 18]. However, an animal model of ECPR reported inconclusive effects of early reperfusion and quick cooling for organ preservation [19]. Whereas, although ECPR with TTM showed significant improvement of outcome, favorable neurological was unsatisfied result (16%), then, usage criteria should be continued to brush up for reducing overuse of ECPR with TTM, which needs more cost and effort. The present study could reveal possibility of improving neurological outcome in ECPR with TTM, however, which could not reach our satisfaction enough.

This study has several limitations. First, although the registry comprises a nationwide cohort, the study was performed retrospectively, which may introduce some bias. Second, the neurological outcomes were assessed in terms of the CPC at 1 month after resuscitation. It is possible that the neurological outcomes might have changed after 6 months or 1 year. Third, while the propensity score analysis demonstrated the efficacy of TTM, other factors might confound the results and introduce some bias. Fourth, the actual body temperatures were not recorded in patients who did not undergo TTM, and it is unknown whether their body temperatures differed from patients who underwent TTM. Fifth, implementation time of ECMO was not shown in database, might have potential to affect the outcome. Sixth, the database did not have the data of interval from collapse to ECMO initiation (inserting catheters), and ECMO initiation to ECMO pump start, these were no considered.

## Conclusions

These analyses of a nationwide Japanese registry showed the possibility that ECPR with TTM could be superior to ECPR without TTM for adult patients with witnessed OHCA.

## Data Availability

The datasets are only available to members of the study group.

## Abbreviations

CCPR: Conventional cardiopulmonary resuscitation
CPC: Glasgow–Pittsburgh Cerebral Performance Category
ECMO: Extracorporeal membrane oxygenation
ECPR: Extracorporeal cardiopulmonary resuscitation
ICPS: Interval from collapse to pump start
ITM: Interval of temperature management
OHCA: Out-of-hospital cardiac arrest
TTM: Target temperature management
VA: Veno-arterial
VF: Ventricular fibrillation
VT: Ventricular tachycardia
